# Evaluation of Customised Lineage-Specific Sets of MIRU-VNTR Loci for Genotyping *Mycobacterium tuberculosis* Complex Isolates in Ghana

**DOI:** 10.1371/journal.pone.0092675

**Published:** 2014-03-25

**Authors:** Adwoa Asante-Poku, Michael Selasi Nyaho, Sonia Borrell, Iñaki Comas, Sebastien Gagneux, Dorothy Yeboah-Manu

**Affiliations:** 1 Bacteriology Department, Noguchi Memorial institute For Medical Research, University of Ghana, Legon, Ghana; 2 Department of Medical Parasitology and Infection Biology, Swiss Tropical and Public Health Institute, Basel, Switzerland; 3 University of Basel, Basel, Switzerland; 4 Biochemistry Department, University of Ghana, Legon, Ghana; 5 Genomics and Health Unit, Centre for Public Health Research, Valencia, Spain; 6 CIBER (Centros de Investigación Biomédica en Red) in Epidemiology and Public Health, Madrid, Spain; St. Petersburg Pasteur Institute, Russian Federation

## Abstract

**Background:**

Different combinations of variable number of tandem repeat (VNTR) loci have been proposed for genotyping *Mycobacterium tuberculosis* complex (MTBC). Existing VNTR schemes show different discriminatory capacity among the six human MTBC lineages. Here, we evaluated the discriminatory power of a “customized MIRU12” loci format proposed previously by Comas *et al*. based on the standard 24 loci defined by Supply *et al*. for VNTR-typing of MTBC in Ghana.

**Method:**

One hundred and fifty-eight MTBC isolates classified into Lineage 4 and Lineage 5 were used to compare a customized lineage-specific panel of 12 MIRU-VNTR loci (“customized MIRU-12″) to the standard MIRU-15 genotyping scheme. The resolution power of each typing method was determined based on the Hunter-Gaston- Discriminatory Index (HGDI). A minimal set of customized MIRU-VNTR loci for typing Lineages 4 (Euro-American) and 5 (*M. africanum* West African 1) strains from Ghana was defined based on the cumulative HGDI.

**Results and Conclusion:**

Among the 106 Lineage 4 strains, the customized MIRU-12 identified a total of 104 distinct genotypes consisting of 2 clusters of 2 isolates each (clustering rate 1.8%), and 102 unique strains while standard MIRU-15 yielded a total of 105 different genotypes, including 1 cluster of 2 isolates (clustering rate: 0.9%) and 104 singletons. Among, 52 Lineage 5 isolates, customized MIRU-12 genotyping defined 51 patterns with 1 cluster of 2 isolates (clustering rate: 0.9%) and 50 unique strains whereas MIRU-15 classified all 52 strains as unique. Cumulative HGDI values for customized MIRU-12 for Lineages 4 and 5 were 0.98 respectively whilst that of standard MIRU-15 was 0.99. A union of loci from the customised MIRU-12 and standard MIRU-15 revealed a set of customized eight highly discriminatory loci: 4052, 2163B, 40, 4165, 2165, 10,16 and 26 with a cumulative HGDI of 0.99 for genotyping Lineage 4 and 5 strains from Ghana.

## Introduction

Tuberculosis (TB) is a major public health problem worldwide, causing 8.8 million new cases and more than 1.4 million deaths each year [1]. The main strategy for controlling TB, especially in low resourced countries, is case detection and treatment using the directly observed treatment short course (DOTS) strategy [2]. The conventional indicators used for assessing TB control programmes focuses on the proportion of patients with new sputum smear positive pulmonary disease that are cured by the end of treatment or whose sputum microscopy becomes negative after the first 2 months of treatment [3]. Such indicators ignore equally important aspects of TB control such as the duration of infectivity, the frequency of reactivation, and the risk of progression among the infected contacts, or the risk of transmission. Thus the control of TB also depends on understanding the patterns and dynamics of transmission which is useful for the implementation of public health measures to reduce sources of infection [4], [5].

A number of molecular markers are available for differentiating members of the *Mycobacterium tuberculosis* complex (MTBC) for conventional epidemiological investigations of TB outbreaks and to assess risk factors associated with recent transmissions [6], [7]. Mycobacterial interspersed repetitive unit-variable number of tandem repeats (MIRU-VNTR) typing, have overcome most of the shortcomings of IS*6110* RFLP [8]–[10], and have now replaced this older technique as the new gold standard for molecular epidemiological investigation of TB. MIRU-VNTR typing which uses genomic diversity at different VNTR loci can have a cumulative resolution comparable to that of IS*6110* RFLP analysis depending on the combination of loci analysed [11]–[17].

Several combinations of MIRU- VNTR loci have been published with initial methods relying on only a few loci, which turned out to have low discriminatory power among MTBC isolates [18], [37]–[39]. Subsequently, a standard MIRU-12 loci set with discriminatory power close to *IS6110*-RFLP was proposed for molecular epidemiological studies in TB [19]–[21]. More recently, this initial MIRU-12 set was replaced by the standard MIRU-15 set, and currently, standard MIRU- 24 loci set [34] has been proposed for optimal discrimination of closely related strains. The standard MIRU15 set which includes six of the previous MIRU- 12 with nine additional loci has been recommended as the standard for routine molecular epidemiology of TB, including outbreak investigations and population-based transmission studies. MIRU-24 set comprises the same 15 loci plus an additional nine provide additional information aimed at phylogenetic and population genetic aspects of MTBC.

The usage of the standard MIRU-15 and MIRU-24 has helped to gain insight into the transmission dynamics of MTBC. However, the initial selection of these loci was to some extent biased towards strains belonging to Lineage 4 (Euro-American lineage) [34]. The inability of the proposed loci led to new customized sets for Lineage 2 strains that include the clinically relevant Beijing family of strains [18]. However, the human-associated MTBC includes 6 additional lineages [22], [23], [45]–[47], which show a strong phylogeographic structure [24]–[26]. As observed for Lineage 2 strains, this might suggest that the usage of high discriminatory MIRU-VNTR loci may be sub-optimal in areas such as Ghana where about 20% of all TB cases are caused by Lineages 5 and 6 of MTBC (also known as *M. africanum* West Africa 1 and 2) [27]–[28].

Comas *et al.* [30] using 108 global MTBC strains [30] showed that the majority of the loci included in standard MIRU-24 had a variable discriminatory power across the different MTBC lineages. Moreover, the MIRU-VNTR loci that exhibited the highest discrimination index within one lineage were not necessarily the ones with the highest discriminatory power in other lineages. Based on the allelic diversity of individual MIRU-VNTR locus, Comas *et al*. [30] suggested different combinations of MIRU-VNTR loci that offered high resolution for the different MTBC lineages. These combinations offered two main advantages over the existing one; it maximized allelic diversity for a given MTBC lineage and allowed for cost effective analyses [30].

Here we evaluated this concept in the Ghanaian setting and compared the standard MIRU-15 to two lineage-specific 12-loci sets (here referred to as “customized MIRU-12”), one for Lineage 4 and one for Lineage 5, which are the most frequent MTBC lineages in Ghana [27]–[28], [49].

## Materials and Methods

### Ethics Statement

Ethical clearance for this study was obtained from the IRB of the Noguchi Memorial Institute for Medical Research, which has a Federal wide Assurance number FWA00001824. The procedure for sampling in this study was basically the same as those outlined by the National Tuberculosis Programme for the routine management of TB in Ghana. Informed consent both written (in the case of literate participants) and oral (for illiterates) was sought from all participants before their inclusion in the study. Consent was sought from their parents or guardians on behalf of children below sixteen years. As per the guidelines of the institutional review board of the Noguchi Memorial Institute for Medical Research, the objectives and benefits of the study were explained to all participants and they were assured of the confidentiality of all information collected from during the study.

### Isolate Selection and Lineage Classification

A total of 178 MTBC isolates consecutively selected from a pool of retrospective samples were included in the study. Specimens included in this study were collected consecutively over a period of 17 months (from October 2007 to March 2009) from sputum AFB-positive pulmonary TB cases attending four main government health centres covering three different regions: Central, Greater Accra and Western regions of Ghana respectively before commencement of anti-TB drug. DNA was extracted as described previously [33]. MTBC was confirmed by *IS6110* PCR [40]. The isolates were then classified into lineages by analyses of various regions of difference (RDs) as previously described [31]. Specifically, all isolates were first screened for RD9. RD9-deleted strains were screened for RD4. Isolates identified as RD9 deleted and RD4 undeleted were further sub-typed for Lineage 5 and 6 (*M. africanum* West Africa I and II) using RD711 and RD702 flanking primers, respectively. TaqMan real time PCR was performed according to standard procedures using probes designed by Stucki *et al* for the confirmation of Lineages [35]. Although Lineage 6 strains (*M. africanum* West Africa II) are present in Ghana [27], [28], they were removed from further analysis due to limited number (6 isolates) identified.

### MIRU-VNTR Analysis

Two sets of PCRs were performed for each isolate. The first set was performed using the 12 lineage-specific MIRU-VNTR loci proposed by Comas *et al*. [30], while the second set consisted of the standard MIRU-15 as described by Supply *et al*. [34] (Table 1). Each PCR mixture contained 10X PCR buffer, 1.5 mM MgCl_2_, 200 μM concentrations of deoxyribonuclueotide triphosphate, 5 μM concentration of each primer, 1 μl of HotstarTaq DNA polymerase enzyme, 5 μl Q solution and 10 ng of DNA template in a total volume of 25 μl. Negative (sterile water) and positive controls (H37Rv) were added to each PCR reaction to validate the assay. Locus amplification was carried out under the following conditions: initial denaturation at 95°C for 15 minutes, and then 40 cycles of 95°C for 1 minute, 59°C for1 minute and 72°C for 3 minutes, followed by a final extension at 72°C for 7 minutes. Gel electrophoresis was done in 2% agarose for 5 hours at 80 constant Voltage. The amplicons were sized using a 100 bp marker and the obtained size compared with allelic table as published by Supply *et al.* [34].

**Table 1:** List of MIRU-VNTRs used for the assay.

LOCUS	ALIAS	15	L4	L5
424	Mtub04	X	X	X
577	ETRC	X	X	X
580	MIRU04	X		
802	MIRU40	X	X	X
960	MIRU10	X	X	X
1644	MIRU16	X		
1955	Mtub21	X	X	X
2163b	QUB11b	X	X	X
2165	ETRA	X	X	X
2401	Mtub30	X	X	
2531	MIRU23		X	X
2996	MIRU26	X		
3007	MIRU27			X
3192	MIRU31	X		X
3690	Mtub39	X	X	
4052	QUB26	X	X	X
4156	QUB4156	X	X	X

### SNP Typing

TaqMan real time PCR was performed as published by Stucki *et al*. [35]. Briefly, in a 200 μl sterile PCR tube, 2 μl of DNA was added to a 5 μl sterile water containing 0.21 μM each reverse and forward primers for the targeted regions, 0.83 μM each probe A for ancestral allele and probe B for mutant allele (each labelled with different dyes); and 5 μl Taqman Universal MasterMix II (Applied Biosystem). The reaction was performed in Applied Biosystems 7300 real time PCR system under the following conditions: 60°C for 30 seconds, 95°C 10 minutess, 95°C 15 seconds and 60°C 1 minute for 40 cycles; 60°C for 30 seconds. The fluorescence intensity in the dyes (VIC and FAM) channels were measured at the end of each cycle.

### Data Analysis

The number of repeats for each locus was determined based on the allelic table by Supply *et al.* [34] and clustering analysis was done using the online tool at http://www.MIRU-VNTRplus.org. MIRU-VNTR clusters were defined as isolates sharing identical patterns. The clustering rate was defined as (*n*c - *c*)/*n*, where *n*c is the total number of clustered cases, *c* is the number of clusters, and *n* is the total number of cases in the sample [29].

The Hunter-Gaston Discriminatory Index (HGDI) was used to calculate the discriminatory power of each locus as well as that of each method [36].

### Determination of a Minimal Set of MIRU-VNTR Loci

Stepwise analysis was performed to identify a set of loci needed to achieve maximum discrimination. Firstly, we combined loci from the customised sets and standard MIRU-15 for each lineage under investigation. Twelve loci were shared between the customised Lineage 4 set and standard MIRU-15, addition of the remaining 4 non-shared loci from standard MIRU-15 gave a total of 16 loci for analysis. For Lineage 5, addition of 6 non-shared loci to the 9 shared loci gave a total of 17 loci. Subsequently, we calculated individual locus HGDI. The results obtained were arranged in a descending order. Starting with the highest HGDI, cumulative HGDI was then calculated by successively adding one locus after the other. Finally, the clustering rate was calculated in a similar manner by successively adding one locus after the other. The result (cumulative HGDI and percentage clustering) obtained for each lineage was plotted on a graph and the cut-off point for selection of the minimal set of loci was set at where graph plateaued meaning further addition of loci resulted in the same cumulative HGDI. The customized minimal loci-set was then extracted from the graph.

## Results

### MTBC Isolates and Lineage Determination

All 178 isolates included in this study were classified into Lineage 4 (N = 126) or Lineage 5 (N = 52) based on the RD and SNP typing analysis [31], [35]. Discordant samples were excluded from the study. A full set of MIRU allelic data was obtained for 158/178 (88.8%), comprising 106 Lineage 4 and 52 Lineage 5 isolates, respectively. The remaining 20 of the 178 (11.2%) isolates were excluded from the analysis for various reasons. 90% (18/20) of excluded isolates had no PCR amplicon at one or several loci whilst the remaining 10% (2/20) had double alleles at one or more MIRU-VNTR loci, indicative of the possible presence of two independent strains [32].

### Evaluation of Customized MIRU-12 for Lineage 4

One hundred and four distinct patterns (Table S1) comprising 102 singleton and 2 clusters (2 isolate per cluster) was obtained using customized MIRU-12 (clustering rate: 1.8%). Discriminatory power was calculated separately for each locus and classified into highly (HGDI ≥0.6), moderately (0.3 to 0.6), and poorly (<0.3) discriminatory based on the HGDI scores as previously reported [21]. Based on this definition, the discriminatory power of 10 loci (MIRU-VNTR loci 10, 40, 2163b, 2165, 3690, 4052, 4165 2401, 0424, and 0577) was higher than 0.6, supporting their designation as highly discriminatory loci with the remaining 2 MIRU/VNTR loci (VNTR 1955, and 23) being “moderately discriminatory”(DI: 0.3–0.59) (Table 2). Using the same set of isolates, standard MIRU-15 identified 105 distinct patterns with only one cluster of two isolates (clustering rate: 0.9%). Ten loci (66.6%; MIRU-VNTR loci 4052, 2163b, 40, 2165, 10, 4165, 3690, 2401, 26 and 0424) were highly discriminatory, 4 (26.6%) (VNTR 1955, 0577 and 23) moderately discriminatory and the remaining 1 (MIRU 4: 6.7%) poorly discriminated among the isolates.

**Table 2:** Cumulative HGI and clustering rate for Lineage 4 successive addition of individual MIRU-VNTR Loci.

Locus	VNTR Loci	Alias	No. of Clusters	No. of clustered isolates	No. of isolates in individual cluster	Clustering Rate (%)	Individual HGI	Cumulative HGI
1	4052	QUB26	9	96	2–34	82.1	0.829	0.829
2	2163B	QUB11B	23	88	2–9	61.3	0.804	0.966
3	802	MIRU40	20	53	2–8	31.1	0.742	0.988
4	2165	ETRA	13	28	2–4	14.2	0.702	0.996
5	960	MIRU10	8	19	2–4	10.4	0.689	0.997
6	4156	QUB4165	4	11	2–4	6.6	0.639	0.999
7	3690	Mtu39	3	6	2	2.8	0.66	0.999
8	2401	Mtu30	3	6	2	2.8	0.64	0.999
9	2996	MIRU26	2	4	2	1.8	0.628	0.999
10	424	Mtu04	2	4	2	1.8	0.613	0.999
11	577	ETRC	2	4	2	1.8	0.553	0.999
12	1995	Mtub21	2	4	2	1.8	0.579	0.999
13	2531	VNTR23	2	4	2	1.8	0.555	0.999
14	1644	MIRU16	2	4	2	1.8	0.452	0.999
15	3192	MIRU31	1	2	2	0.9	0.37	0.999
16	580	MIRU4	1	2	2	0.9	0.092	0.999

(N = 106).

### Evaluation of Customized MIRU-12 for Lineage 5

Among 52 isolates analyzed, we obtained 51 unique patterns with 1 cluster (Table S1) comprising 2 isolates and a clustering rate of 0.9%. Five MIRU-VNTR loci (2163B, 4156, 4052, 40, 27) were highly discriminatory and the remaining 7 (23, 0577, 2165, 10, 0424, 31, 1955) being moderately discriminatory (Table 3). Standard MIRU-15 on the other hand identified 52 unique patterns. Six of the 15 loci (VNTR 2163B, 4156, 4052, 26, 16 and 40) had HGDI above 0.6 with the remaining nine MIRU-VNTR loci (0424, 10, 1955, 0577, 4, 2401, 3690, 2165 and 31) showing moderate discrimination (HGDI: 0.3–0.59).

**Table 3:** Cumulative HGI and clustering rate for Lineage 5 with successive addition of individual MIRU-VNTR Loci.

Locus	VNTR Loci	Alias	No. of Clusters	No. of clustered isolates	No. of isolates in individual cluster	Clustering Rate (%)	Individual HGI	Cumulative HGI
1	2163B	QUB11B	6	52	2–13	88.5	0.797	0.798
2	4156	QUB4156	12	45	2–9	63.5	0.731	0.935
3	802	MIRU 40	2	4	2	3.8	0.7	0.971
4	2996	MIRU 26	7	16	2–3	15.9	0.7	0.992
5	4052	QUB26	10	31	2–5	40.4	0.69	0.997
6	1644	MIRU 16	3	6	2	5.7	0.689	0.998
7	3007	MIRU 27	2	4	2	3.8	0.6	0.998
8	2531	MIRU 23	2	4	2	3.8	0.58	0.998
9	424	Mtub04	2	4	2	3.8	0.572	0.998
10	960	MIRU 10	2	4	2	3.8	0.526	0.998
11	1955	Mtub21	2	4	2	3.8	0.489	0.998
12	577	ETRC	2	4	2	3.8	0.487	0.998
13	2401	Mtub30	1	2	2	1.9	0.473	0.998
14	580	MIRU04	1	2	2	1.9	0.472	0.999
15	3690	Mtub39	0	0	0	0	0.428	0.999
16	2165	ETRA	0	0	0	0	0.387	0.999
17	3192	MIRU31	0	0	0	0	0.352	0.999

N = 52.

### Determination of a Minimal Set of MIRU-VNTR Loci for Genotyping Main MTBC Lineages from Ghana

**Figure 1:**
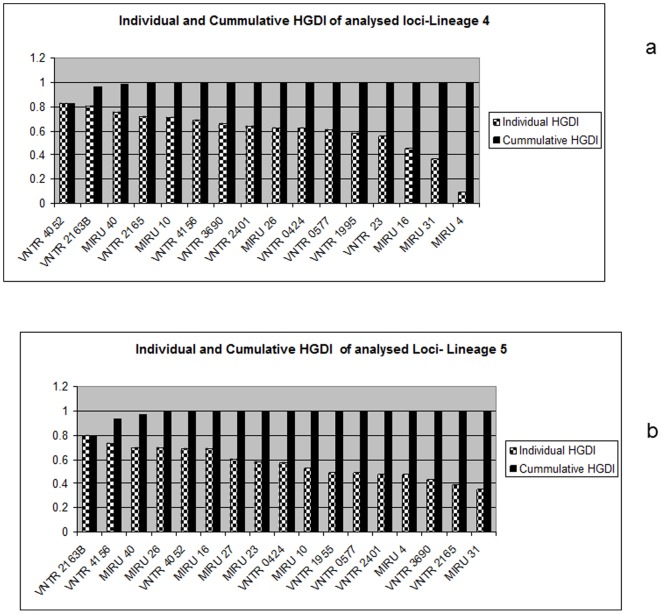
Individual and Cumulative HGI of analysed Loci. Individual and cumulative HGI was calculated for each locus and after successive loci addition respectively for all analysed loci using the mathematical formula as proposed by Hunter and Gaston (36). The black bars are the cumulative HGI values while crossed bars are for the individual locus values. Fig. 1a and 1b show values for lineage 4 and 5 respectively.

Customized MIRU-12 for Lineage 4 shared 11 loci with standard MIRU-15 whilst 9 loci were shared between customized MIRU-12 for Lineage 5 and standard MIRU-15. A union of both sets of typing schemes gave a total of 16 and 17 loci for Lineage 4 and 5, respectively (Table S1). For Lineage 4, we identified six top most discriminatory loci (4052, 2163B, 40, 2165, 10 and 4165) with a cumulative HGDI of 0.99 (Table 2). Similarly, for Lineage 5, six loci: 2163B, 4165, 40, 26, 4052 and 16 (Table 3) with a cumulative HGDI of 0.99 were identified. Further addition of loci gave no significant change in cumulative HGDI values as shown in Figure 1. Note that 4 loci (4052, 2163B, 4162 and 40) were among the 6 most discriminatory in both lineage-specific sets. Hence, based on this, we propose the usage of a new set of customised typing system comprising 8 loci showing the highest discriminatory power for genotyping strains from the two most common lineages circulating in Ghana.

## Discussion and Conclusion

Different combinations of MIRU and other VNTR loci have been proposed to complement the standard MIRU-15 scheme to achieve higher discrimination. Results accumulated from such studies clearly revealed that due to the strong phylogeographic structure exhibited by MTBC, the most relevant MIRU-VNTR typing schemes will likely differ depending on the specific geographical setting. For example, Shamputa *et al.* [18] successfully identified a reduced set of 8 loci from standard MIRU-24, which could be used to discriminate, isolates from the Republic of Korea. Similarly, Musare *et al.* [37], Dong *et al.* [38] and Zhou *et al.* [39] successfully defined a minimal set of 12 loci for genotyping Beijing strains which made up more than 90% of the isolates investigated from Asia. Most of the studies have been focused on Lineage 2 including the clinically important Beijing family based on its association with drug resistance [48]. However, no study has been carried out in most resource-limited settings like Ghana, where *M. africanum* is an important pathogen [27]–[28], [49]. If customized lineage-specific sets of MIRU-VNTR loci could be implemented in such settings, this will have an impact in terms of reducing work load and saving resources. In the present study, we evaluated such an approach for genotyping MTBC strains from Ghana, [27], [28] and compared our results with the current gold standard typing method; standard MIRU 15 as proposed by Supply *et al.* [34].

**Figure 2:**
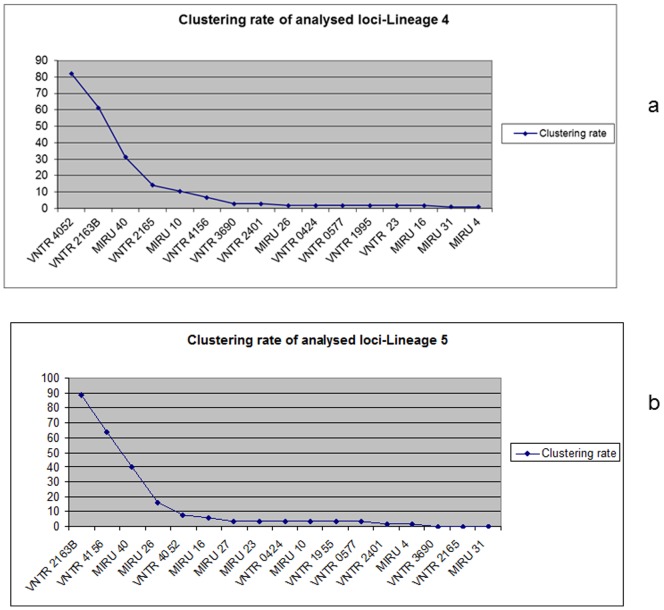
Clustering rate for lineages 4 and 5 calculated using after successive addition of analysed loci using the formula (*n*c - *c*)/*n*, where *n*c is the total number of clustered cases, *c* is the number of clusters, and *n* is the total number of cases in the sample a was calculated after successive addition of individual locus. Fig. 2a and b shows clustering rate values for lineages 4 and 5 respectively.

Although standard MIRU-15 showed higher discrimination in its ability to accurately identify clusters among these two lineages in our study when compared to customised lineage-specific MIRU-12 proposed previously [30], we found that not all the 15 loci were as informative for typing MTBC strains in Ghana. Even with the customized MIRU-12, based on our data, not all 12 loci were needed to achieve maximum discrimination (Figure 1). Specifically, our analysis showed that 10 of a total of 16 loci tested for Lineage 4 strains added no or only limited additional information in terms of discriminatory power. Similarly, 11 of a total of 17 loci screened for Lineage 5 strains showed limited discriminatory power. We thus explored the possibility of a minimal set of loci HGDI selected by combining the standard MIRU-15 and the customized MIRU-12 data set. Based on individual and cumulative HGDIs, and clustering rate, we defined the six top discriminatory loci for Lineage 4 (4052, 2163B, 40, 2165, 10 and 4165) (Figure 1a and 2a) and similarly for Lineage 5 (2163B, 4165, 40, 26, 4052 and 16) (Figure 1b and 2b) with 4 loci shared among the two sets (4052, 2163B, 40 and 4165). A combination of loci from Lineage 4 and 5 gave a unique customized set of 8 loci with HGDIs similar to that of standard MIRU-15.

We now plan to apply these minimal MIRU-VNTR set for molecular epidemiological investigation of MTBC transmission in population based study in Ghana. We anticipate that this approach will save a significant amount of time. In addition we perform cost analysis on the different VNTR schemes analysed in this study. Cost was calculated based on the direct cost of reagents, materials and equipment. We compared the cost of genotyping using standard MIRU-15 and our proposed customized set of MIRU-8. With a unit cost of $11.24, the cost of performing standard MIRU-15 on one sample was $168.60, with the total material costs of analyses using our proposed customized MIRU-8 set for one sample being $89.2. Hence, by screening for only the relevant loci, we not only maximize discriminatory power but also minimize genotyping costs.

Currently, human-associated MTBC is known to comprise a total of seven main phylogenetic lineages [23], [46]–[47]. We propose that additional lineage-specific sets of MIRU-VNTR could be identified for molecular epidemiological investigation of TB transmission in resource-limited settings. Moreover, each MTBC lineage consists of a number of sub-lineages, some of which also show strong geographical associations [22, 24, and 45]. For example, the “Uganda” sub-lineage of Lineage 4 causes up to 60% of TB in Kampala, Uganda [41], suggesting that a similar customized Lineage 4 set for Uganda could be developed,which possibly would include other loci considering that most of Lineage 4 in Ghana consists of the “Cameroon” sub-lineage [42]–[44].

This study set out to define a set of loci for genotyping MTBC strains from Ghana. We acknowledge the high prevalence of *M. africanum* strains in Ghana, however, this prevalence is driven by Lineage 5 (*M. africanum* West Africa I) with limited number of Lineage 6 (*M. africanum* West Africa II). We acknowledge the fact that this makes our proposed customized MIRU-8 country-specific, and thus suggest that countries within West African where the high prevalence of *M. africanum* is driven by Lineage 6 (*M. africanum* West Africa II) could equally determine the minimal set of loci which gives the highest discrimination. Nevertheless, the strength of our study is the ability to genotype an unknown strain in Ghana with the proposed customized MIRU-8 loci in the most cost-efficient way.

In conclusion, this study identified a reduced set which can be applied for strain differentiation of the main MTBC lineages from Ghana.

## Supporting Information

Table S1 MIRU-VNTR Profiles of the Mycobacterial Isolates Analysed.
(XLS)
